# Incidence and risk factors for enteral nutrition intolerance in psychiatric patients with comorbid medical conditions: a retrospective cohort study

**DOI:** 10.3389/fpsyt.2026.1904251

**Published:** 2026-10-05

**Authors:** Pei Zhou, Dongyan Zhang, Ping Zhang, Jiajia Shi, Xiaomin Liu, Shujuan Jiao, Beibei Wang, Xia Xue

**Affiliations:** 1 Department of Critical Care Medicine, Nantong Fourth People’s Hospital, Nantong, Jiangsu, China; 2 Hemodialysis Center, Nantong Fourth People’s Hospital, Nantong, Jiangsu, China

**Keywords:** critical illness, enteral nutrition intolerance, physical comorbidities, psychiatric patients, retrospective cohort study, risk factors

## Abstract

**Background:**

Critically ill psychiatric patients frequently present with multiple physical comorbidities and poor nutritional status. Enteral nutrition plays a key role in comprehensive critical care, but enteral nutrition intolerance (ENI) is highly prevalent.

**Aims:**

To investigate the incidence, independent risk factors, and clinical outcomes of ENI in critically ill psychiatric patients with comorbid physical diseases.

**Methods:**

This is a single-center retrospective study. We enrolled 150 consecutive patients admitted between January 2024 and December 2025 who received enteral nutrition for at least 3 days. Data for analysis were sourced from the institutional electronic medical record database. Baseline characteristics, nutritional indicators, psychiatric parameters, comorbidity scores, and treatment-related variables were collected. To detect independent risk factors for ENI, univariate screening was performed first, followed by multivariate logistic regression.

**Results:**

The overall incidence of ENI was 54.0% in critically ill psychiatric patients with physical comorbidities. Multivariate logistic regression showed the odds ratio (OR) of serum albumin <25 g/L (OR = 3.26, 95%CI: 2.11–5.04, P<0.001), APACHE II score ≥15 (OR = 2.89, 95%CI: 1.87–4.47, P<0.001), combined use of ≥3 antipsychotic agents (OR = 2.71, 95%CI: 1.75–4.19, P<0.001), schizophrenia (OR = 2.12, 95%CI: 1.43–3.21, P = 0.008), and duration of antibiotic use ≥7 days (OR = 2.18, 95%CI: 1.40–3.38, P = 0.001). ENI was significantly associated with longer hospital stay, lower nutritional goal attainment, more frequent EN interruption, and higher pulmonary infection rates.

**Conclusion:**

These findings indicate a high burden of ENI in this vulnerable population. The risk factors include hypoalbuminemia, high APACHE IIscore, combined use of three or more antipsychotic agents, schizophrenia, and prolonged antibiotic therapy.

## 1 Introduction

Critically ill psychiatric patients, including those with severe schizophrenia, major depressive disorder, and acute bipolar disorder, frequently suffer from multiple physical comorbidities. The proportion was reported to be as high as 25% (1). These include cardiovascular diseases, diabetes, chronic obstructive pulmonary disease, gastrointestinal disorders, and so on (2–4). Poor lifestyle, side effects of psychotropic medications, metabolic abnormalities, and severe psychiatric symptoms contribute to poor nutritional status in this population (5, 6).

Enteral nutrition is a core component of comprehensive treatment for critically ill patients (7). It improves nutritional condition, enhances immune function, promotes recovery of physical and psychiatric symptoms, and reduces adverse outcomes. However, enteral nutrition intolerance (ENI) is a common complication during enteral nutrition support, with a reported incidence of 65.5% (8). Typical manifestations include diarrhea, constipation, abdominal distension, and upper GI intolerance (8, 9). ENI impairs the efficacy of nutritional support, increases infection risk, prolongs hospital stay, raises medical costs, and worsens patient prognosis (10, 11).

However, research specifically targeting critically ill psychiatric patients with physical comorbidities remains limited. This population has unique characteristics, including impaired consciousness, extremely poor compliance, severe eating abnormalities, and more prominent gastrointestinal side effects of psychotropic drugs. Thus, the risk of ENI may be significantly higher than in other patient groups, and its risk factors may be specific. The present research sought to evaluate the incidence of ENI and identify related risk factors in this special population. The results are supposed to provide evidence for the prevention and intervention of ENI in critically ill psychiatric patients with physical comorbidities.

## 2 Methods

### 2.1 Study design

This was a retrospective cohort study conducted in a specialized intensive psychiatric care unit. Consecutive patients admitted between Jan 2024 and Dec 2025 were screened. Patients were identified using the electronic medical record system. This system contains all the demographic, medical and nursing data of patients. This study was approved by the Clinical Research Ethics Committee of Nantong Fourth People’s Hospital with waiver of informed consent (No. 2026-L002). This manuscript was prepared using the STROBE guidelines for reporting observational studies.

### 2.2 Study population

Patients were eligible if they met all of the following criteria: (1) diagnosis of severe mental disorders, including severe schizophrenia, bipolar disorder, or major depressive disorder, in accordance with the Diagnostic and Statistical Manual of Mental Disorders, Fifth Edition (DSM-5) or the International Classification of Diseases, 11th Revision (ICD-11); (2) critical psychiatric status requiring intensive monitoring; (3) at least one physical comorbidity confirmed by clinical examination, laboratory tests, or imaging studies; (4) enteral nutrition support administered for ≥ 3 consecutive days; (5) complete electronic medical records available for analysis.

Patients meeting any of the following criteria were excluded from the study: (1) absolute contraindications to enteral nutrition; (2) exclusive parenteral nutrition without enteral nutrition; (3) severe cognitive or communication impairment that precluded reliable assessment of gastrointestinal symptoms; (4) missing rate of key clinical indicators ≥ 20%; (5) transfer to other hospitals or early withdrawal during enteral nutrition support.

### 2.3 Data collection

Retrospective extraction of clinical data from electronic medical records was conducted by two independent trained researchers. Two trained researchers independently extracted not only baseline covariates but also all clinical records related to ENI-relevant manifestations, including diarrhea frequency, vomiting/abdominal distension records, gastric residual volume measurements, and enteral nutrition interruption events. After independent extraction, all ENI-related outcome judgements were cross-compared. Any discrepancies in ENI case identification were submitted to a senior investigator for final adjudication to resolve disagreements. All patient-identifiable information was de-identified before data extraction and analysis to protect participant privacy.

Collected variables included: (1) demographic characteristics, such as age, sex, body mass index (BMI), smoking history, and alcohol consumption; (2) psychiatric parameters, including diagnosis, disease duration, severity of psychiatric symptoms, number and dosage of antipsychotic agents, and use of sedative-hypnotic medications; (3) physical comorbidities: number of somatic diseases, cases of gastrointestinal dysmotility due to various reasons, Acute Physiology and Chronic Health Evaluation II (APACHE II) score, Charlson Comorbidity Index (CCI), acute exacerbation of physical diseases, severe infection, and mechanical ventilation; (4) nutritional status: Nutrition Risk Screening 2002 (NRS 2002) score, serum albumin, prealbumin, hemoglobin, and serum electrolytes; (5) enteral nutrition -related parameters: formula type, initial infusion rate, daily volume, feeding route, and rapid rate escalation; (6) other variables: duration of antibiotic therapy, length of hospital stay, occurrence and manifestations of enteral nutrition intolerance (ENI).

### 2.4 Definition of enteral nutrition intolerance

The diagnosis of enteral nutrition intolerance was based on one or more of the following manifestations during enteral infusion, which could not be explained by other organic diseases and improved after slowing the infusion rate, changing the formula, or temporary interruption: (1) diarrhea: ≥ 3 loose or watery bowel movements per day for more than 24 hours; (2) vomiting or abdominal distension: ≥ 1 episode of obvious vomiting per day or significant abdominal distension with increased abdominal girth; (3) gastric retention: gastric residual volume ≥ 200 mL on two consecutive measurements or ≥ 300 mL on a single measurement; (4) severe gastrointestinal symptoms leading to permanent interruption of enteral nutrition.

### 2.5 Definition of clinical outcomes and key variables

Nutritional goal attainment: Achievement of ≥80% of the prescribed daily enteral nutrition volume and calorie target for 3 consecutive days, without persistent feeding intolerance or unplanned interruption.

Pulmonary infection: Diagnosed based on new or progressive pulmonary infiltrates on chest imaging plus at least two of the following: fever (body temperature ≥38.5°C), purulent sputum, leukocytosis (white blood cell count >10×10^9^/L) or leukopenia (white blood cell count <4×10^9^/L), and positive pathogenic evidence from sputum culture or bronchoalveolar lavage fluid.

Acute exacerbation of physical diseases: Rapid worsening of pre-existing chronic physical comorbidities requiring emergency treatment, increased monitoring, or escalation of medical interventions within 24 hours.

Severe infection: Evidence of systemic infection accompanied by organ dysfunction or failure, including sepsis, severe pneumonia, bloodstream infection, urinary tract infection with shock, or other life-threatening infectious conditions, as defined by ICU admission criteria and Sepsis-3 criteria.

### 2.6 Statistical analysis

All statistical analyses were performed using SPSS 26.0 software (IBM Corp., Armonk, NY, USA). Continuous variables were tested for normality using the Shapiro-Wilk test. Normally distributed data were presented as mean ± standard deviation (SD) and compared using the independent-samples t-test. Non-normally distributed data were expressed as median (interquartile range, IQR) and analyzed using the Wilcoxon rank-sum test. Categorical variables were reported as numbers (percentages) and compared using the chi-square test or Fisher exact test. Subgroup analysis was performed according to the primary psychiatric diagnosis (schizophrenia, bipolar disorder, and major depressive disorder).

Univariate analysis was initially performed to screen potential risk factors for ENI. Variables with a P value < 0.10 in univariate analysis were included in the multivariate logistic regression model to identify independent risk factors for ENI. The cut-off values for continuous variables included in the primary multivariate regression analysis were determined by receiver operating characteristic (ROC) curve analysis according to the maximum Youden index. Considering that this data-driven dichotomization approach may inflate type-I error risk, two sets of sensitivity analyses were further performed to test the robustness of the findings. On the one hand, key continuous variables (serum albumin, APACHE II score, duration of antibiotic use) were directly entered into the multivariate model without dichotomization. On the other hand, pre-specified clinically meaningful thresholds were applied: serum albumin < 25 g/L, APACHE II score ≥ 15, and duration of antibiotic use ≥ 7 days. Collinearity diagnostics were performed to exclude significant multicollinearity between variables. Hosmer-Lemeshow goodness-of-fit test and calibration plot were used to assess the calibration performance of the final multivariate logistic regression model. Results were expressed as odds ratios (OR) with corresponding 95% confidence intervals (CI). A two-sided P < 0.05 was considered statistically significant.

To address potential confounding from non-unified enteral nutrition protocols and psychotropic medication adjustments, all baseline and treatment-related variables (feeding route, initial rate, antibiotic duration, number of antipsychotics, sedative use) were included in univariate screening and controlled in the multivariate logistic regression model. Collinearity diagnostics were performed to ensure robustness. Stratified analyses were also conducted to evaluate consistency across subgroups.

The missing rate of key variables (demographics, nutritional indicators, psychiatric parameters, comorbidity scores, and treatment variables) was below 5% in the final cohort, with no systematic missing pattern observed. We used complete-case analysis for all statistical procedures. Given the low proportion of missing data, complete-case analysis was considered appropriate and unlikely to introduce substantial bias; multiple imputation was not performed.

### 2.7 Sample size and power analysis

This was a single-center retrospective cohort study enrolling all consecutive eligible patients admitted during the study period. A formal prospective sample size calculation was not performed prior to data collection, as this was an observational, real-world study based on available clinical data.

For the multivariate logistic regression model, we followed the widely accepted 10 events per variable (EPV) rule. The primary outcome was enteral nutrition intolerance (ENI), which occurred in 81 patients. We included 5 independent risk factors in the final model; thus, the number of events (81) was sufficient relative to the number of variables (5), satisfying the EPV criterion and ensuring statistical stability.

A *post-hoc* power analysis was performed using the observed incidence of ENI (54.0%), an α level of 0.05 (two-sided), and the number of predictors. The analysis achieved a statistical power > 80%, indicating that the sample size of 150 patients was adequate to detect the identified independent risk factors for ENI.

## 3 Results

As shown in **Figure 1**, a total of 150 critically ill psychiatric patients with comorbid physical diseases who received enteral nutrition were enrolled in this study. The overall incidence of ENI was 54.0% (81/150). Patients were divided into the ENI group (n=81) and non-ENI group (n=69). Among the 81 patients with ENI, the most common clinical manifestations were diarrhea in 59 patients (72.8%), vomiting or abdominal distension in 47 patients (58.0%), gastric retention in 38 patients (46.9%), and aspiration pneumonia in 9 patients (11.1%).

**Figure 1:**
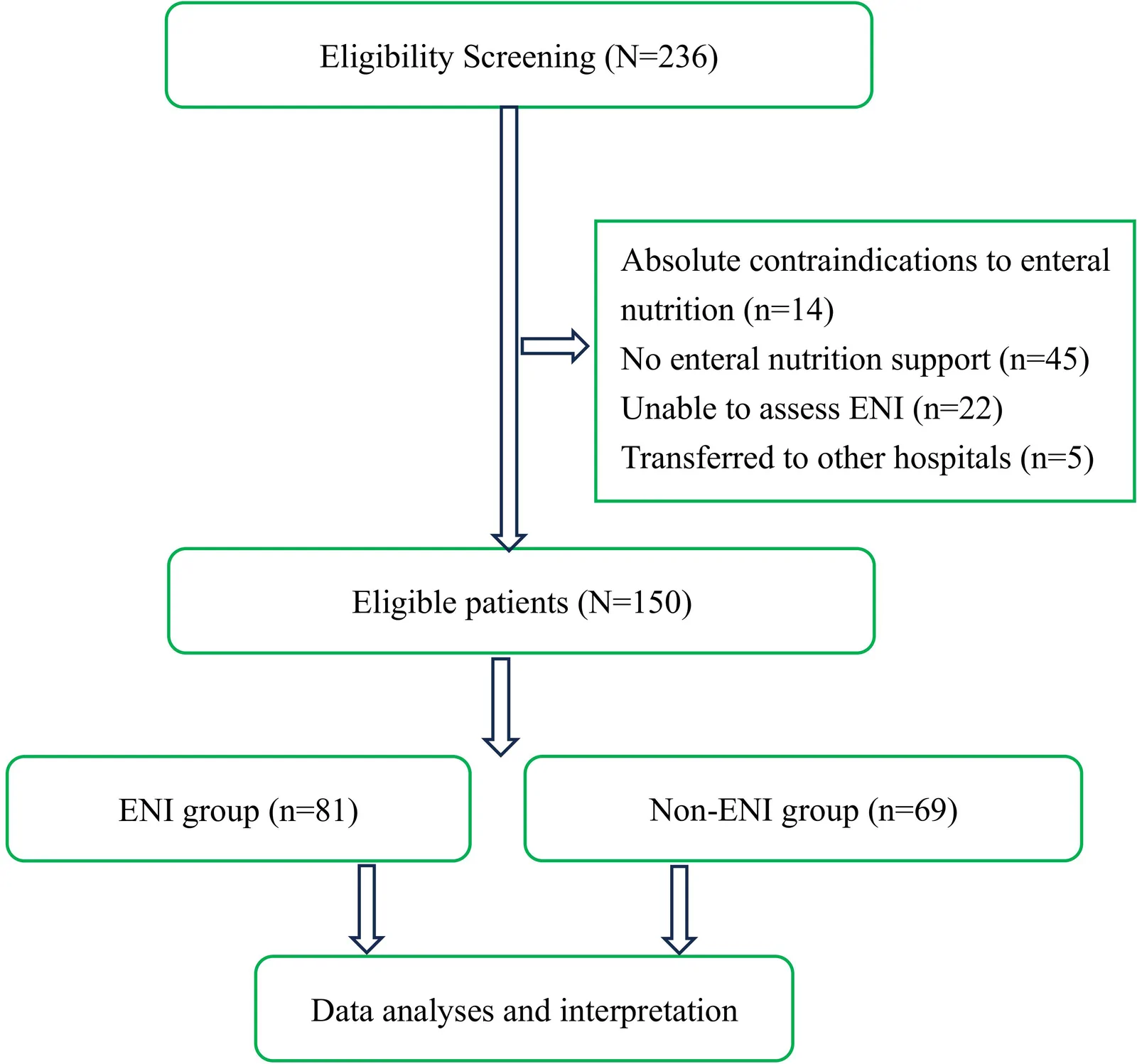
Study flowchart. ENI, enteral nutrition intolerance.

**Table 1** presents the baseline comparisons. The results showed that patients in the ENI group were significantly older (62.1 ± 7.8 vs. 50.6 ± 8.2 years, P<0.001), had lower BMI (18.1 ± 1.9 vs. 22.7 ± 2.3 kg/m², P<0.001), longer duration of mental illness (14.2 ± 4.8 vs. 8.3 ± 4.1 years, P<0.001), higher APACHE II scores (21.8 ± 3.7 vs. 13.5 ± 3.2, P<0.001), higher CCI scores (6.2 ± 1.5 vs. 3.4 ± 1.3, P<0.001), and higher NRS 2002 scores (6.1 ± 1.1 vs. 3.7 ± 1.0, P<0.001) than those in the non-ENI group. Serum albumin (25.2 ± 6.8 vs. 31.6 ± 3.1 g/L, P<0.001) and prealbumin (121.3 ± 22.6 vs. 182.5 ± 25.7 mg/L, P<0.001) were significantly lower in the ENI group. In addition, the initial enteral nutrition infusion rate was faster (38.7 ± 6.4 vs. 32.11 ± 5.2 ml/h, P<0.001) and the duration of antibiotic use was longer (11.7 ± 3.2 vs. 4.3 ± 2.1 days, P<0.001) in the ENI group. However, the number of patients with gastrointestinal dysmotility between the two groups were comparable (10 (12.3%) vs. 5 (7.2%), P = 0.169).

**Table 1:** Baseline characteristics and clinical data of patients with and without ENI.

Variables	Total (n=150)	ENI group (n=81)	Non-ENI group (n=69)	p
Age, years	56.8 ± 9.7	62.1 ± 7.8	50.6 ± 8.2	<0.001
Male, n (%)	82 (54.7)	48 (59.3)	34 (49.3)	0.232
BMI, kg/m²	20.4 ± 2.6	18.1 ± 1.9	22.7 ± 2.3	<0.001
Smoking, n (%)	63 (42.0)	39 (48.1)	24 (34.8)	0.113
Drinking, n (%)	72 (48.0)	45 (55.6)	27 (39.1)	0.049
Psychiatric diagnosis, n (%)				<0.001
Schizophrenia	75 (50.0)	54 (66.7)	21 (30.4)	
Bipolar disorder	48 (32.0)	21 (25.9)	27 (39.1)	
Major depression	27 (18.0)	6 (7.4)	21 (30.4)	
Duration of mental illness, years	11.5 ± 5.2	14.2 ± 4.8	8.3 ± 4.1	<0.001
PANSS/HAMD score	76.3 ± 18.5	89.5 ± 10.2	61.7 ± 12.4	–
Number of antipsychotics, n (%)				<0.001
1	36 (24.0)	6 (7.4)	30 (43.5)	
2	48 (32.0)	21 (25.9)	27 (39.1)	
≥3	66 (44.0)	54 (66.7)	12 (17.4)	
Sedative-hypnotic use, n (%)	96 (64.0)	69 (85.2)	27 (39.1)	<0.001
Number of physical diseases, n (%)				<0.001
1-2	63 (42.0)	15 (18.5)	48 (69.6)	
≥3	87 (58.0)	66 (81.5)	21 (30.4)	
Gastrointestinal dysmotility, n (%)	15 (10.0)	10 (12.3)	5 (7.2)	0.169
Severe infection, n (%)	78 (52.0)	57 (70.4)	21 (30.4)	<0.001
Mechanical ventilation, n (%)	69 (46.0)	54 (66.7)	15 (21.7)	<0.001
NRS 2002 score	4.9 ± 1.5	6.1 ± 1.1	3.7 ± 1.0	<0.001
Albumin, g/L	27.4 ± 4.2	25.2 ± 6.8	31.6 ± 3.1	<0.001
Prealbumin, mg/L	152.6 ± 31.4	121.3 ± 22.6	182.5 ± 25.7	<0.001
Hemoglobin, g/L	108.4 ± 12.7	101.2 ± 9.8	116.8 ± 11.3	<0.001
Potassium, mmol/L	3.6 ± 0.4	3.4 ± 0.3	3.8 ± 0.4	<0.001
Initial infusion rate, ml/h	35.6 ± 8.2	38.7 ± 6.4	32.11 ± 5.2	<0.001
Enteral nutrition rapid escalation, n (%)	84 (56.0)	69 (85.2)	15 (21.7)	<0.001
Feeding route, n (%)				<0.001
Nasogastric tube	105 (70.0)	69 (85.2)	36 (52.2)	
Nasojejunal tube	45 (30.0)	12 (14.8)	33 (47.8)	
Duration of antibiotics, days	8.2 ± 4.1	11.7 ± 3.2	4.3 ± 2.1	<0.001

ENI, enteral nutrition intolerance; BMI, body mass index; PANSS, Positive and Negative Syndrome Scale; HAMD, Hamilton Depression Rating Scale; APACHE II, Acute Physiology and Chronic Health Evaluation II; CCI, Charlson Comorbidity Index; NRS 2002, Nutrition Risk Screening 2002.

In addition, subgroup analysis was performed according to the type of primary psychiatric disorder. Among the 150 patients, 75 had schizophrenia, 48 had bipolar disorder, and 27 had major depressive disorder. The incidences of ENI in patients with schizophrenia, bipolar disorder, and major depressive disorders were 72.0%, 43.8%, and 22.2%, respectively. There was a statistically significant difference in the incidence of ENI across the three psychiatric subgroups (P < 0.001). Differences among the three groups were statistically significant. The schizophrenia group had older age, poorer nutritional status, more complex medication regimens, more severe comorbidities, and higher rates of acute exacerbation and infection (all P < 0.05) (**Table 2**).

**Table 2:** Comparisons among the patients with schizophrenia, bipolar disorder, and major depression.

Variables	Schizophrenia (n=75)	Bipolar disorder (n=48)	Major depression (n=27)	p
Age, years	62.5 ± 7.2	51.6 ± 11.7	48.7 ± 2.9	<0.001
Male, n (%)	43 (57.3)	21 (43.8)	18 (66.7)	0.102
BMI, kg/m²	18.6 ± 1.6	21.2 ± 3.1	22.2 ± 0.7	<0.001
Smoking, n (%)	28 (37.3)	20 (41.7)	15 (55.6)	0.258
Drinking, n (%)	30 (40.0)	25 (52.1)	17 (63.0)	0.041
Duration of mental illness, years	15.5 ± 5.4	8.9 ± 5.9	7.1 ± 1.8	<0.001
Number of antipsychotics, n (%)				<0.001
1	12 (16.0)	13 (27.1)	11 (40.7)	
2	20 (26.7)	16 (33.3)	12 (44.4)	
≥3	43 (57.3)	19 (39.6)	4 (14.8)	
Sedative-hypnotic use, n (%)	57 (76.0)	21 (43.8)	18 (66.7)	<0.001
Number of physical diseases, n (%)				0.016
1-2	28 (37.3)	17 (35.4)	18 (66.7)	
≥3	47 (62.7)	31 (64.6)	9 (33.3)	
Gastrointestinal dysmotility, n (%)	7 (9.3)	5 (10.4)	3 (11.1)	0.911
APACHE IIscore	20.8 ± 4.4	13.7 ± 5.6	11.5 ± 1.4	<0.001
CCI score	6.5 ± 2.1	3.8 ± 2.1	3.2 ± 0.6	<0.001
Acute exacerbation, n (%)	66 (88.0)	17 (35.4)	10 (37.0)	<0.001
Severe infection, n (%)	48 (64.0)	18 (37.5)	12 (44.4)	<0.001
Mechanical ventilation, n (%)	40 (58.7)	21 (43.8)	8 (29.6)	0.003
NRS 2002 score	6.8 ± 1.3	4.5 ± 1.8	3.9 ± 0.6	<0.001
Albumin, g/L	24.8 ± 6.6	30.4 ± 4.8	33.6 ± 3.1	<0.001
Prealbumin, mg/L	125.4 ± 21.4	170.9 ± 37.5	185.4 ± 7.1	<0.001
Hemoglobin, g/L	91.0 ± 6.7	113.9 ± 9.8	122.3 ± 11.3	<0.001
Potassium, mmol/L	3.2 ± 0.4	3.8 ± 1.8	4.0 ± 1.4	<0.001
Initial infusion rate, ml/h	38.1 ± 4.9	37.0 ± 5.3	38.2 ± 7.6	0.108
Enteral nutrition rapid escalation, n (%)	54 (72.0)	17 (35.4)	13 (48.1)	<0.001
Feeding route, n (%)				0.584
Nasogastric tube	55 (73.3)	33 (68.8)	17 (63.0)	
Nasojejunal tube	20 (26.7)	15 (31.2)	10 (37.0)	
Duration of antibiotics, days	12.0 ± 4.1	6.7 ± 3.2	4.3 ± 2.1	<0.001

ENI, enteral nutrition intolerance; BMI, body mass index; PANSS, Positive and Negative Syndrome Scale; HAMD, Hamilton Depression Rating Scale; APACHE II, Acute Physiology and Chronic Health Evaluation II; CCI, Charlson Comorbidity Index; NRS 2002, Nutrition Risk Screening 2002.

Univariate analysis indicated that age ≥60 years, BMI <18.5 kg/m², schizophrenia diagnosis, use of ≥3 antipsychotics, sedative-hypnotic drug use, ≥3 physical comorbidities, severe infection, acute exacerbation of physical diseases, mechanical ventilation, NRS 2002 ≥5, serum albumin <25 g/L, nasogastric tube feeding, rapid infusion escalation, and antibiotic use ≥7 days were potential risk factors for ENI (all P<0.1).

Multivariate logistic regression analysis further identified five independent risk factors for ENI: serum albumin <25 g/L (OR = 3.26, 95%CI: 2.11–5.04, P<0.001), APACHE II score ≥15 (OR = 2.89, 95%CI: 1.87–4.47, P<0.001), combined use of ≥3 antipsychotic agents (OR = 2.71, 95%CI: 1.75–4.19, P<0.001), schizophrenia (OR = 2.12, 95%CI: 1.43–3.21, P = 0.008), and duration of antibiotic use ≥7 days (OR = 2.18, 95%CI: 1.40–3.38, P = 0.001) (**Table 3**). No significant differences in the direction of risk effects were observed among the subgroups. Collinearity diagnostics were performed for all variables included in the multivariate logistic regression model. The variance inflation factor (VIF) of each variable ranged from 1.03 to 1.42, and all tolerance values were greater than 0.60. No significant multicollinearity was detected among the independent variables.

**Table 3:** Multivariate logistic regression analysis of independent risk factors for ENI.

Risk factors	OR (95%CI)	P
Serum albumin <25 g/L	3.26 (2.11–5.04)	<0.001
APACHE IIscore ≥15	2.89 (1.87–4.47)	<0.001
≥3 antipsychotics combined	2.71 (1.75–4.19)	<0.001
Higher initial enteral nutrition infusion rate	1.10 (0.84–1.16)	0.278
Duration of antibiotics ≥7 days	2.18 (1.40–3.38)	0.001
Age ≥60 years	1.24 (0.76–2.02)	0.382
BMI <18.5 kg/m²	1.21 (0.76–1.91)	0.422
Schizophrenia diagnosis	2.12 (1.43–3.21)	0.008
≥3 physical comorbidities	1.26 (0.77–2.06)	0.352
Severe infection	1.19 (0.74–1.92)	0.471
Mechanical ventilation	1.21 (0.74–1.98)	0.442
NRS 2002 ≥5	1.18 (0.74–1.89)	0.481
Nasogastric tube feeding	1.25 (0.77–2.04)	0.358
Rapid infusion escalation	1.24 (0.76–2.01)	0.385

ENI, enteral nutrition intolerance; OR, odds ratio; CI, confidence interval; APACHE II, Acute Physiology and Chronic Health Evaluation II; BMI, body mass index; NRS 2002, Nutrition Risk Screening 2002. The cut-off values are based on ROC curve analysis.

When key predictors were included as continuous variables in multivariate logistic regression, the direction and statistical significance of risk-factor associations were consistent with the primary analysis. Sensitivity analysis adopting pre-defined clinical thresholds also reproduced the same independent risk factors with comparable OR magnitudes. The Hosmer-Lemeshow test showed satisfactory model calibration (χ^2^ = 7.42, P = 0.492).

With respect to clinical outcomes (**Figure 2**), significant associations were observed between ENI and longer hospital stay (18.5 ± 5.2 vs. 12.3 ± 4.6 days, P<0.001), lower rate of nutritional goal attainment (56.8% vs. 84.1%, P<0.001), higher rate of enteral nutrition interruption or adjustment (69.1% vs. 21.7%, P<0.001), and higher incidence of pulmonary infection (28.4% vs. 7.2%, P<0.001), when comparing patients with versus without ENI.

**Figure 2:**
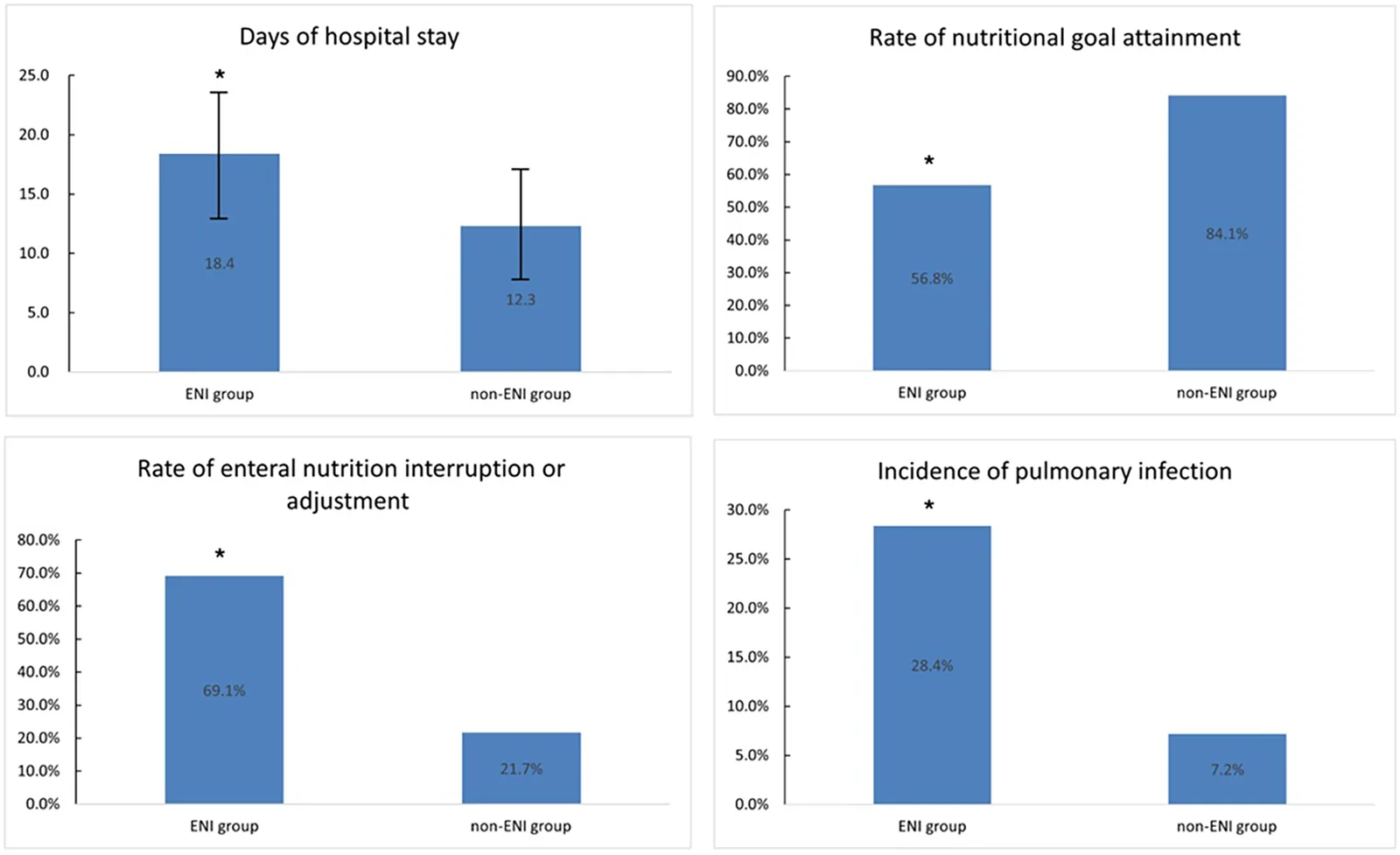
Clinical outcomes of patients in ENI group and non-ENI group. ENI, enteral nutrition intolerance. *P<0.05 in comparison with non-ENI group.

## 4 Discussion

This retrospective cohort study investigated the incidence, risk factors, and clinical impacts of ENI in critically ill psychiatric patients with comorbid physical diseases. The results indicated that the incidence of ENI reached 54.0% in this population; low serum albumin, high APACHE IIscore, combined use of three or more antipsychotic agents, schizophrenia, and prolonged antibiotic administration were independent risk factors for ENI; and ENI was significantly associated with prolonged hospital stay, lower nutritional goal attainment, higher enteral nutrition interruption or adjustment rate, and increased pulmonary infection.

In the present study, the overall incidence of ENI in critically ill psychiatric patients was 54.0%. Previous studies reported that the incidence of ENI ranged from 29% to 65% (8, 12). Our finding falls within this range but is relatively high, which may be attributed to the unique clinical characteristics of critically ill psychiatric patients. First, this population often suffers from severe psychiatric symptoms, impaired consciousness, poor compliance, and restricted activity, all of which predispose to delayed gastric emptying and gastrointestinal dysmotility. Second, long-term use of multiple antipsychotic agents and sedative-hypnotic drugs exerts inhibitory effects on gastrointestinal peristalsis, further increasing the risk of feeding intolerance (13–15). Third, the high prevalence of malnutrition, multiple physical comorbidities, and critical illness severity in our cohort also contributes to compromised gut function and higher ENI risk.

Notably, subgroup analysis according to psychiatric diagnosis demonstrated that the incidence of ENI was significantly higher in patients with schizophrenia than in those with bipolar disorder or major depressive disorder. Several reasons may account for this observation. Patients with schizophrenia often have longer disease duration, more severe psychiatric symptoms, and poorer self-care ability, all of which contribute to chronic malnutrition, hypoalbuminemia, and impaired gastrointestinal function (16, 17). In addition, patients with schizophrenia tend to have more physical comorbidities, higher disease severity, and longer periods of sedative-hypnotic use, further reducing enteral nutrition tolerance. However, the explanation for the differences among the three groups could not be determined due to the retrospective design of this study.

Common risk factors for ENI in critically ill patients can be summarized into the following three main categories (12, 18). Patient-related factors include advanced age, low body mass index, high APACHE II score, hypoproteinemia, hypokalemia, sepsis, organ dysfunction, and underlying digestive system diseases. Nutrition-related factors mainly involve delayed initiation of enteral nutrition, rapid infusion rate, lack of dietary fiber, and non-post-pyloric feeding. In addition, treatment-related factors such as sedative drugs, vasoactive agents, use of multiple antibiotics, mechanical ventilation, elevated intra-abdominal pressure, and high central venous pressure also significantly increase the risk of feeding intolerance. In the present study, we investigated the risk factors for ENI in critically ill psychiatric patients. The results indicated that serum albumin <25 g/L, APACHE II score ≥15, combined use of ≥3 antipsychotic agents, and duration of antibiotic use ≥7 days were closely associated with ENI in critically ill psychiatric patients with comorbid physical diseases.

Hypoalbuminemia is a well-established marker of malnutrition and critical illness (19, 20). Low albumin level impairs intestinal mucosal integrity, reduces intestinal blood perfusion, delays gastric emptying, and weakens gastrointestinal motility, all of which contribute to feeding intolerance. It was reported that patients with serum albumin levels below 2 g/dL showed a significantly higher rate of diarrhea than those with albumin above 2 g/dL (27.0% vs. 10.5%) (21). In addition, chronic hypoalbuminemia was associated with a significantly higher diarrhea rate compared with acute-onset hypoalbuminemia (35.1% vs. 9.9%) (21). Early nutritional assessment and timely correction of hypoalbuminemia before or at the initiation of enteral nutrition may help reduce the occurrence of ENI and improve the clinical outcomes (22).

ENI was significantly associated with adverse clinical outcomes, such as longer duration of mechanical ventilation and ICU hospitalization, and higher mortality hazard ratio (11, 23). In the present study, patients with ENI had longer hospital stay, lower nutritional goal attainment rate, higher EN interruption or adjustment rate, and higher pulmonary infection rate. Significant associations between ENI and unfavorable clinical endpoints were identified in the present cohort. Such observed correlations may reflect complex interplay among nutritional delivery status, immune function, aspiration risk and nosocomial infection. In addition, it is thought that poor nutrition may cause low mood, while improving diet may protect both physical and mental health (24, 25). These findings emphasize that preventing and managing ENI is critical for improving clinical outcomes in critically ill psychiatric patients with physical comorbidities.

Several limitations of this study should be acknowledged. First, given the single center retrospective design, complete elimination of residual confounding originating from non-protocolized enteral nutrition strategies and variable psychotropic medication adjustment is not feasible. Although key treatment-related variables were adjusted for in multivariate regression, unmeasured or incompletely captured clinical practice differences may still bias our effect estimates. Second, some detailed information was unavailable, such as specific drug names and doses, gastrointestinal motility monitoring data, and intestinal microbiological results, which hinders in-depth exploration of the mechanisms underlying ENI. Furthermore, as ENI diagnosis was retrospectively determined based on documented clinical manifestations rather than objective gastrointestinal function tests, ascertainment bias and inter-observer variability originating from chart-record completeness cannot be fully excluded, even with dual independent extraction and adjudication procedures. Variations in clinical documentation practice among bedside clinicians may also affect ENI case recognition. Moreover, long-term follow-up of patients after discharge was lacking, making it impossible to evaluate the long-term impact of ENI on nutritional status, mental symptom recovery, and long-term mortality. In addition, enteral nutrition protocols and psychotropic medication adjustment strategies were not fully unified among patients, which may affect the comparability between groups. Finally, the study only included patients with severe schizophrenia, bipolar disorder, and major depressive disorder, excluding other types of severe mental illnesses, resulting in limited sample representativeness.

## 5 Conclusion

The incidence of ENI is high in critically ill psychiatric patients with comorbid physical diseases. Independent risk factors of ENI in these patients include hypoalbuminemia, high APACHE IIscore, combined use of three or more antipsychotic agents, schizophrenia, and prolonged antibiotic therapy. ENI is significantly associated with prolonged hospital stay and adverse clinical outcomes.

## Data Availability

The raw data supporting the conclusions of this article will be made available by the authors, without undue reservation.

## References

[1] Halstead S Cao C Høgnason Mohr G Ebdrup BH Pillinger T McCutcheon RA . Prevalence of multimorbidity in people with and without severe mental illness: a systematic review and meta-analysis. Lancet Psychiatry. (2024) 11:431–42. doi: 10.1016/s2215-0366(24)00091-9 38642560

[2] Launders N Hayes JF Price G Osborn DP . Clustering of physical health multimorbidity in people with severe mental illness: An accumulated prevalence analysis of United Kingdom primary care data. PloS Med. (2022) 19:e1003976. doi: 10.1371/journal.pmed.1003976 35442948 PMC9067697

[3] Wouters MM Boeckxstaens GE . Is there a causal link between psychological disorders and functional gastrointestinal disorders? Expert Rev Gastroenterol Hepatol. (2016) 10:5–8. doi: 10.1586/17474124.2016.1109446 26569404

[4] Koloski N Holtmann G Talley NJ . Is there a causal link between psychological disorders and functional gastrointestinal disorders? Expert Rev Gastroenterol Hepatol. (2020) 14:1047–59. doi: 10.1080/17474124.2020.1801414 32715790

[5] Teasdale SB Latimer G Byron A Schuldt V Pizzinga J Plain J . Expanding collaborative care: integrating the role of dietitians and nutrition interventions in services for people with mental illness. Australas Psychiatry. (2018) 26:47–9. doi: 10.1177/1039856217726690 28869391

[6] Vakili O Motamed M Taghizadeh M Khatami SH . Nutritional management of schizophrenia: gut-brain axis mechanisms and dietary interventions. Mol Neurobiol. (2026) 63:460. doi: 10.1007/s12035-026-05751-w 41712175

[7] Al-Dorzi HM Arabi YM . Nutrition support for critically ill patients. JPEN J Parenter Enteral Nutr. (2021) 45:47–59. doi: 10.1002/jpen.2228 34897737

[8] Shi L Shao J Luo Y Liu G OuYang M . The incidence and risk factors of gastrointestinal dysfunction during enteral nutrition in mechanically ventilated critically ill patients. Nurs Open. (2024) 11:e2247. doi: 10.1002/nop2.2247 39474999 PMC11523002

[9] Reintam Blaser A Deane AM Preiser JC Arabi YM Jakob SM . Enteral feeding intolerance: updates in definitions and pathophysiology. Nutr Clin Pract. (2021) 36:40–9. doi: 10.1002/ncp.10599 33242218

[10] Lavrentieva A Kontakiotis T Bitzani M . Enteral nutrition intolerance in critically ill septic burn patients. J Burn Care Res. (2014) 35:313–8. doi: 10.1097/bcr.0b013e3182a22403 24879397

[11] Heyland DK Ortiz A Stoppe C Patel JJ Yeh DD Dukes G . Incidence, risk factors, and clinical consequence of enteral feeding intolerance in the mechanically ventilated critically ill: an analysis of a multicenter, multiyear database. Crit Care Med. (2021) 49:49–59. doi: 10.1097/ccm.0000000000004712 33148950

[12] Xu J Shi W Xie L Xu J Bian L . Feeding intolerance in critically ill patients with enteral nutrition: A meta-analysis and systematic review. J Crit Care Med (Targu Mures). (2024) 10:7–15. doi: 10.2478/jccm-2024-0007 39108795 PMC11193960

[13] Every-Palmer S Lentle RG Reynolds G Hulls C Chambers P Dunn H . Spatiotemporal mapping techniques show clozapine impairs neurogenic and myogenic patterns of activity in the colon of the rabbit in a dose-dependent manner. Front Pharmacol. (2017) 8:209. doi: 10.3389/fphar.2017.00209 28484390 PMC5401895

[14] Palmer SE McLean RM Ellis PM Harrison-Woolrych M . Life-threatening clozapine-induced gastrointestinal hypomotility: an analysis of 102 cases. J Clin Psychiatry. (2008) 69:759–68. 10.4088/jcp.v69n0509 18452342

[15] Yang T Zhou Y Wang M Zhang L Liu B Sun L . Effects of remimazolam tosilate on gastrointestinal hormones and gastrointestinal motility in patients undergoing gastrointestinal endoscopy with sedation: a randomized control trial. Postgrad Med J. (2024) 100:635–41. doi: 10.1093/postmj/qgae040 38538569

[16] Ryan PM . Epidemiology, etiology, diagnosis, and treatment of schizophrenia. Am J Hosp Pharm. (1991) 48:1271–80. doi: 10.1093/ajhp/48.6.1271 1677528

[17] Harris A . Approach to schizophrenia. Intern Med J. (2023) 53:473–80. doi: 10.1111/imj.16068 37070777

[18] Wang S He Y Yi J Sha L . Risk factors for enteral feeding intolerance in critically ill patients: an updated systematic review and meta-analysis. BMC Gastroenterol. (2025) 25:233. doi: 10.1186/s12876-025-03837-8 40200147 PMC11980324

[19] Ventura JC Oliveira LDA Silveira TT Hauschild DB Mehta NM Moreno YMF . Admission factors associated with nutritional status deterioration and prolonged pediatric intensive care unit stay in critically ill children: PICU-ScREEN multicenter study. JPEN J Parenter Enteral Nutr. (2022) 46:330–8. doi: 10.1002/jpen.2116 33818806

[20] Haruki S Minami Y Hattori H Kametani M Im J Oyabu K . Modified nutrition risk in critically ill (mNUTRIC) score on admission and mortality in acute heart failure patients admitted to the cardiac intensive care unit. Heart Vessels. (2026) 41:96–103. doi: 10.1007/s00380-025-02595-7 40797116

[21] Hwang TL Lue MC Nee YJ Jan YY Chen MF . The incidence of diarrhea in patients with hypoalbuminemia due to acute or chronic malnutrition during enteral feeding. Am J Gastroenterol. (1994) 89:376–8. 8122648

[22] Al-Shebli H Al-Shayyab S Younes MNB Badwan S Obeidat M Alshawabkeh A . The clinical impacts of early using glutamine/arginine enriched high protein density formula at trophic dose in intolerant enteral nutrition cachectic hypoalbuminemic hospitalized patients. Rom J Intern Med. (2020) 58:153–60. doi: 10.2478/rjim-2020-0014 32449700

[23] Mehta NM Bechard LJ Cahill N Wang M Day A Duggan CP . Nutritional practices and their relationship to clinical outcomes in critically ill children--an international multicenter cohort study*. Crit Care Med. (2012) 40:2204–11. doi: 10.1097/ccm.0b013e31824e18a8 22564954 PMC3704225

[24] Firth J Gangwisch JE Borisini A Wootton RE Mayer EA . Food and mood: how do diet and nutrition affect mental wellbeing? Bmj. (2020) 369:m2382. doi: 10.1136/bmj.m2382 32601102 PMC7322666

[25] Taylor AM Holscher HD . A review of dietary and microbial connections to depression, anxiety, and stress. Nutr Neurosci. (2020) 23:237–50. doi: 10.1080/1028415x.2018.1493808 29985786

